# Juvenile Dermatomyositis: A Case Report and Review of Literature

**DOI:** 10.7759/cureus.3935

**Published:** 2019-01-22

**Authors:** Pooja Gupta, Sharma Shruti, Vishnu Chaudhary, Geeti Khullar, Fouzia Siraj

**Affiliations:** 1 Pathology, National Institute of Pathology, New Delhi, IND; 2 Dermatology, Safdarjung Hospital, New Delhi, IND

**Keywords:** juvenile dermatomyositis, proximal myopathy, heliotrope rash, gottron papule, calcinosis cutis

## Abstract

Juvenile dermatomyositis (JDM) is a systemic inflammatory disease involving children, which primarily affects the skin and the musculoskeletal system. The characteristic findings include Gottron papules, heliotrope rash, calcinosis cutis, and symmetric proximal muscle weakness. Histologically, it is characterized by the presence of lymphocytic vascular inflammation and endothelial swelling. Herein, we report a case of a 10-year-old girl of Indian origin, who presented to us with classical clinical and histological features of JDM.

## Introduction

Juvenile dermatomyositis (JDM) is a rare multisystemic autoimmune disease of uncertain origin, characterized by chronic inflammation of striated muscles and skin, resulting in rash and proximal muscle weakness [1]. It is reported at an annual incidence of two to four cases per million children per year [2]. This disease has various clinical manifestations, with Gottron papules and heliotrope rash being pathognomic. Calcinosis, which is the deposition of calcium salts in the skin, subcutaneous tissues or the muscles, is often seen as a late sequel of this disorder. The etiopathogenesis of JDM involves a complex interplay of environmental triggers, immune dysfunction, and specific tissue responses. Both cellular and humoral immune-mediated destruction of the vasculature, skin, and the muscles are believed to be involved in its pathogenesis. We report the case of a 10-year-old female child who presented to us with classical clinical and histological features of JDM.

## Case presentation

**Figure 1:**
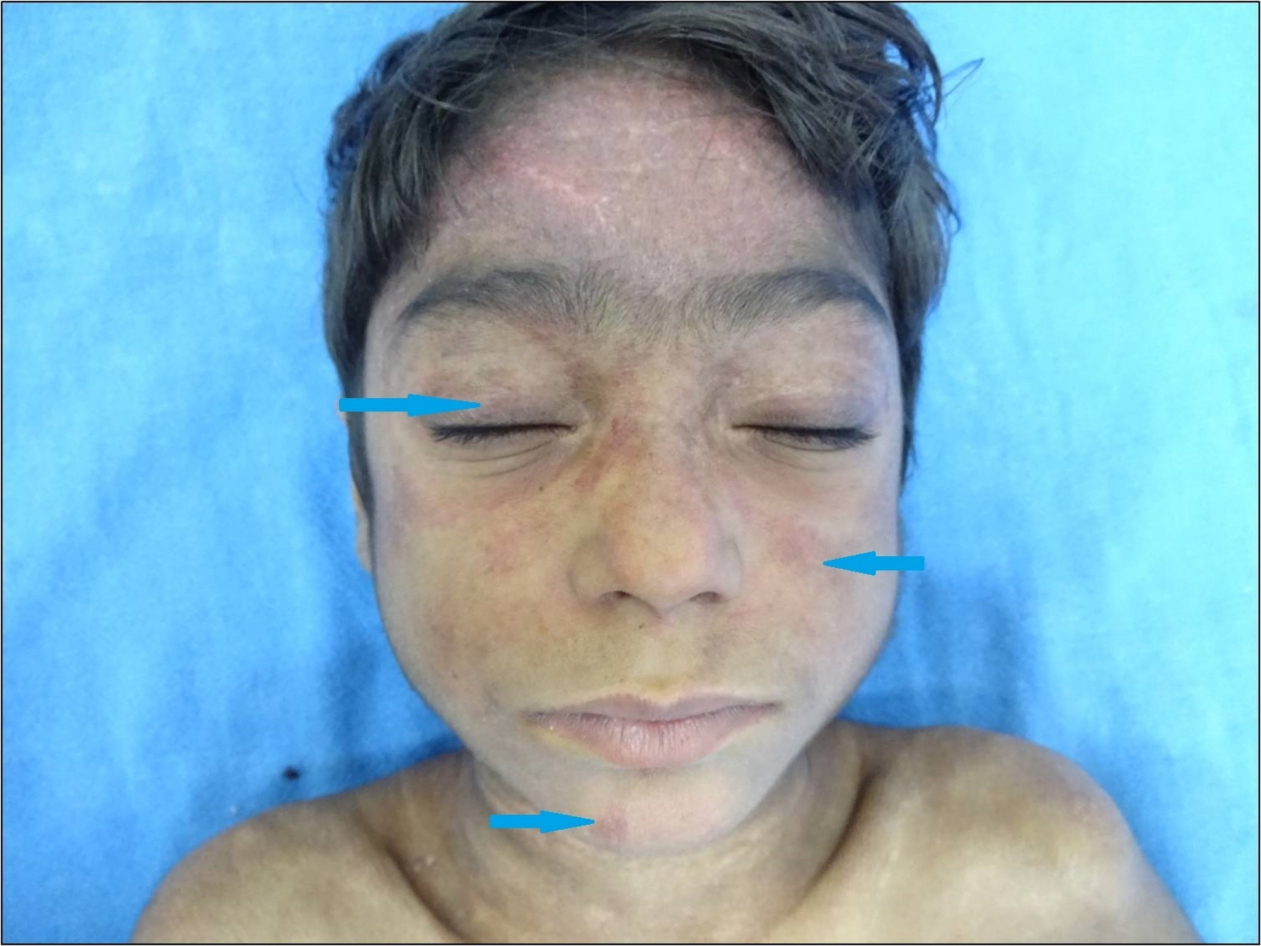
Heliotrope rash Heliotrope rash involving the upper eyelid, cheek, and the chin (arrows)

A 10-year-old girl of Indian origin presented to the pediatric outpatient department with a history of insidious onset and gradually progressive pain and weakness, predominantly affecting the proximal muscles of both upper and lower limbs, for the past four years. She also complained of dusky red rash with swelling, itching, and photosensitivity over the face and extremities, and pain and swelling over bilateral knee joints since the past three years. She also developed multiple hard ulcerated lesions with chalky white discharge over face, chest, trunk, and extremities over the last six months. Her past medical and family history were unremarkable. On general physical examination, the child appeared to be emaciated with a weight of 18 kg (less than the fifth percentile for age), a height of 120 cm (less than the fifth percentile for age), and a body mass index (BMI) of 12.5 kg/m^2^. Examination of skin revealed the presence of confluent violaceous, edematous macules around eyelids, forehead, cheek, and chin (heliotrope rash), and erythematous firm papules of size 0.5 X 0.5 cm over metacarpophalangeal, proximal interphalangeal, and distal interphalangeal joints (Gottron papules;Figure 1).

**Figure 2:**
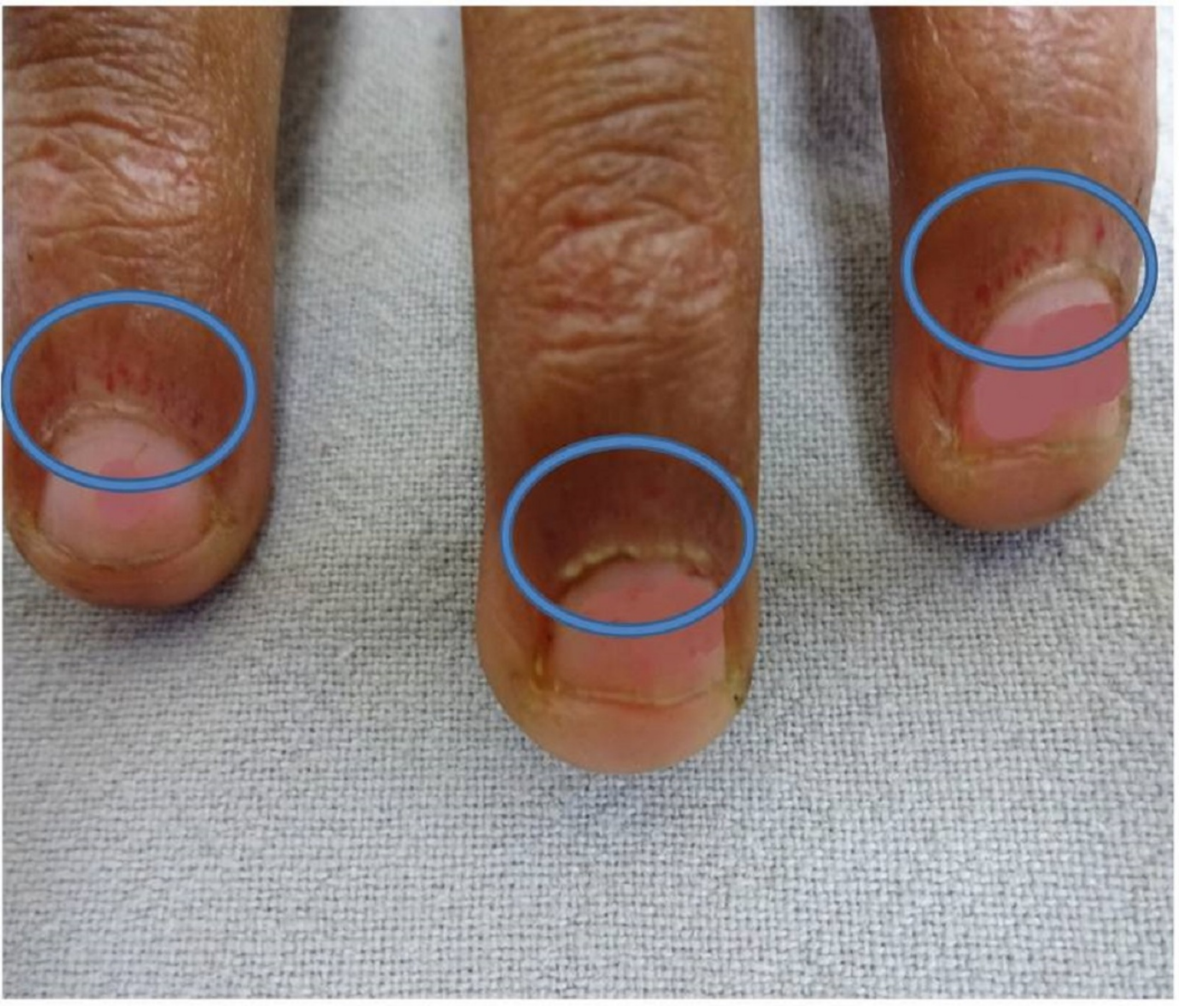
Positive Samitz sign Dystrophic and ragged cuticles (circles)

She also had erythematous scaly plaques over the elbow and the knee joints and hypertrichosis and hyperpigmentation over the forehead, neck, and hands with a dystrophic and ragged cuticle (Samitz sign; Figure 2).

**Figure 3:**
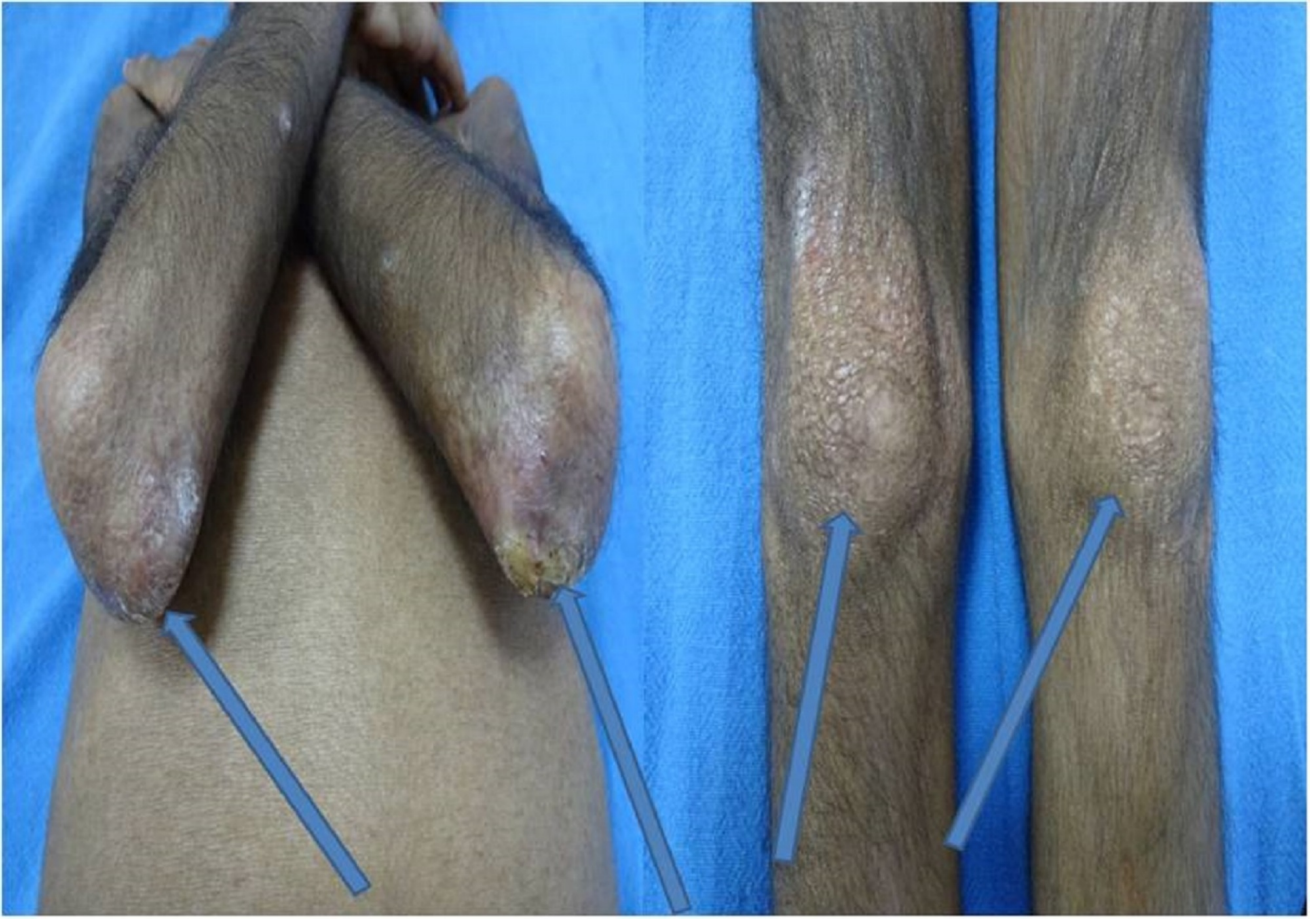
Calcinosis cutis Calcinosis cutis involving bilateral elbows and knees (arrows)

Multiple tender ulcerated subcutaneous nodules measuring 0.5 x 0.5 cm to 1 x 1 cm over bilateral elbows, knees, and trunk were also noted (calcinosis cutis; Figure 3).

On nail capillaroscopy, dilated and tortuous capillaries and capillary dropouts were noted. Musculoskeletal examination revealed minimal tenderness of the proximal muscles of upper and lower limbs with positive Gower’s sign (use of hands and arms to "walk" up the body from squatting position due to proximal myopathy affecting lower limbs). Muscle power in proximal muscles of both upper and lower limbs was 3/5.

**Figure 4:**
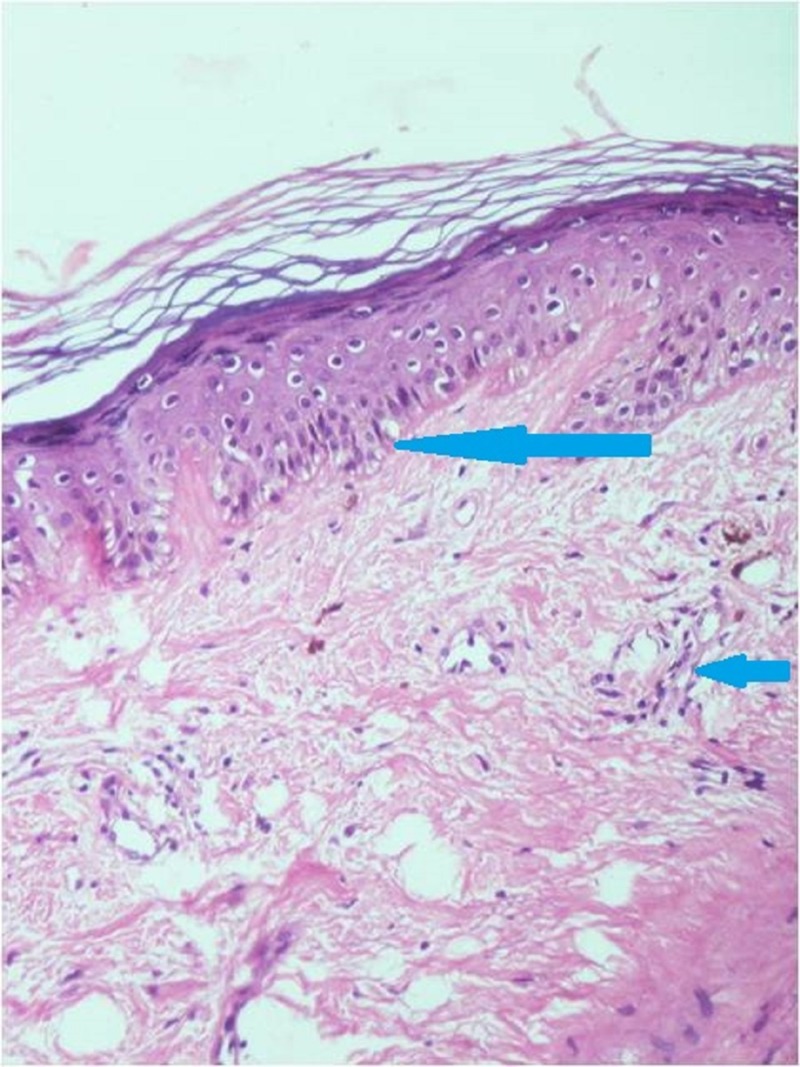
Skin histopathology (hematoxylin and eosin, 200X) Microphotograph showing vacuolar degeneration of basal layer (long arrow) and perivascular lymphohistiocytic infiltration (short arrow)

On evaluation, her liver function tests, renal function tests, fasting plasma glucose, and serum electrolytes were within normal limits. She was found to have microcytic hypochromic anemia with hemoglobin of 9.6 g/dL. Test for antinuclear antibody (ANA) was negative, while lactate dehydrogenase (LDH) level was elevated at 825 U/L (N: 130-240 U/L). Chest radiograph and two-dimensional echocardiogram were also normal. Magnetic resonance imaging (MRI) of the hip, shoulder, and ankle region revealed multiple areas of subcutaneous and intramuscular calcifications. Electromyography (EMG) was suggestive of a myopathic pattern. A punch biopsy from the ulcerated lesion on the forehead was performed, which on histopathology revealed hyperkeratosis, acanthosis and follicular plugging in the epidermis. Mild vacuolar alteration at the dermo-epidermal junction was noted. Upper dermis showed mild edema along with moderate perivascular lymphocytic infiltration (Figure 4).

**Figure 5:**
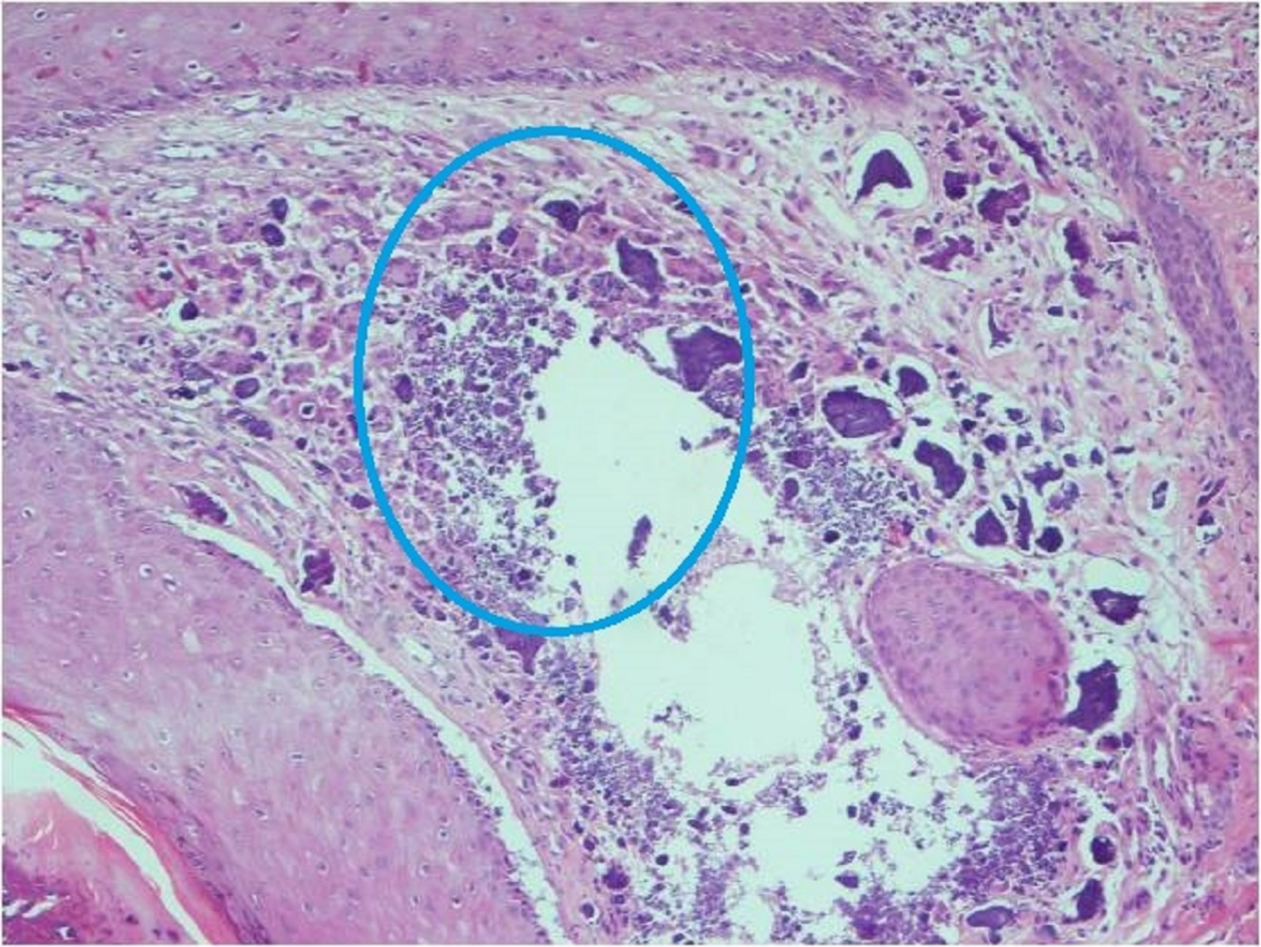
Skin histopathology (hematoxylin and eosin, 100X) Microphotograph showing calcium deposition along with foreign body giant cell reaction in the dermis (circle)

Entire dermis showed a marked dense calcium deposition along with foreign body giant cell reaction (Figure 5).

Taking into account the clinical, biochemical, and histopathological features, the girl was diagnosed with JDM. She was initiated on treatment with two immunomodulator drugs- prednisolone and methotrexate, along with supportive treatment for muscle weakness, rash, and calcinosis cutis.

## Discussion

JDM is a rare autoimmune connective tissue disorder, which is classified as a part of a heterogeneous group of muscle diseases called idiopathic inflammatory myopathies. The precise etiology of this disorder is not clear; however, both immune dysfunction and environmental factors are thought to contribute to its etiopathogenesis. This disorder primarily affects the skin and the striated muscles, resulting in a characteristic rash and proximal muscle weakness.

The diagnosis of JDM is made using the “Bohan and Peter criteria” developed in 1975. The diagnostic criteria include characteristic skin rash, symmetric muscle weakness of the upper and lower proximal muscles, increased levels of serum muscle enzymes, myopathic electromyography, and characteristic pathologic changes revealed by a muscle biopsy [3]. The case presented above fulfilled all the components of the diagnostic criteria for JDM. The yield of diagnostic criteria can be improved further by incorporating new techniques, such as MRI and ultrasonography [4].

The long-term complications of JDM include prolonged and severe muscle weakness with muscle atrophy, cutaneous calcifications, scarring or atrophy, and lipodystrophy [5]. Calcinosis cutis, a form of dystrophic calcification, is a late sequel of the disease and occurs in up to 40% of patients with this disorder. It is a major cause of morbidity and has been linked to younger age at disease onset, persistent disease activity and the presence of anti-NXP2 autoantibodies [6]. A recent addition to the group of myositis-specific autoantibodies is anti-p155/140 antibody, which has been described in 22% to 29% of patients with JDM, 13% to 21% of patients with adult-onset dermatomyositis, and most patients with malignancy-associated dermatomyositis [7-11]. The presence of this antibody in patients with JDM has been shown to correlate with a clinical phenotype comprising of severe cutaneous manifestations [12]. Our patient had a similar clinical phenotype; however, the antibody level could not be measured due to non-availability of this test at our center. 

The most frequently affected sites with calcinosis include elbows, knees, trunk, hands, feet, buttocks, and head; however, any body part may be affected [13]. Calcinosis is most commonly seen at one to three years after the onset of illness; however, it has been reported to occur even at 20 years after the disease onset [14]. Histopathology, as in our case, reveals the presence of foreign body giant cell reaction in the vicinity of calcium deposits along with fibroblasts, lymphocytes, plasma cells, and few eosinophilic infiltrate in the dermis [15].

Although there are no established protocols for the management of patients with JDM, there is enough evidence to suggest that early aggressive management improves outcome, whereas delayed treatment is associated with poor outcomes and higher rates of complications [16]. Since the introduction of corticosteroids, significant improvement in clinical and functional outcome has been achieved and hence, they remain the mainstay of the treatment [17].

## Conclusions

To conclude, we have presented the case of a young girl with classical clinical and histopathological findings of a rare autoimmune disorder. It is important to diagnose and initiate immunosuppressive treatment early in the natural history of the disease in order to reduce the burden of long-term complications. This case has been reported for its rarity and to emphasize the importance of early and aggressive treatment to prevent the long-term disease sequels, which were unfortunately seen in our patient.
